# Comparative Analyses of Pandemic H1N1 and Seasonal H1N1, H3N2, and Influenza B Infections Depict Distinct Clinical Pictures in Ferrets

**DOI:** 10.1371/journal.pone.0027512

**Published:** 2011-11-16

**Authors:** Stephen S. H. Huang, David Banner, Yuan Fang, Derek C. K. Ng, Thirumagal Kanagasabai, David J. Kelvin, Alyson A. Kelvin

**Affiliations:** 1 Division of Experimental Therapeutics, Toronto General Research Institute, University Health Network, Toronto, Ontario, Canada; 2 Department of Immunology, Faculty of Medicine, University of Toronto, Toronto, Ontario, Canada; 3 Immune Diagnostics and Research, Toronto, Ontario, Canada; 4 International Institute of Infection and Immunity, Shantou University Medical College, Shantou, Guangdong, China; 5 Dipartimento di Scienze Biomediche, Universita' degli Studi di Sassari, Sassari, Sardinia, Italia; University of Georgia, United States of America

## Abstract

Influenza A and B infections are a worldwide health concern to both humans and animals. High genetic evolution rates of the influenza virus allow the constant emergence of new strains and cause illness variation. Since human influenza infections are often complicated by secondary factors such as age and underlying medical conditions, strain or subtype specific clinical features are difficult to assess. Here we infected ferrets with 13 currently circulating influenza strains (including strains of pandemic 2009 H1N1 [H1N1pdm] and seasonal A/H1N1, A/H3N2, and B viruses). The clinical parameters were measured daily for 14 days in stable environmental conditions to compare clinical characteristics. We found that H1N1pdm strains had a more severe physiological impact than all season strains where pandemic A/California/07/2009 was the most clinically pathogenic pandemic strain. The most serious illness among seasonal A/H1N1 and A/H3N2 groups was caused by A/Solomon Islands/03/2006 and A/Perth/16/2009, respectively. Among the 13 studied strains, B/Hubei-Wujiagang/158/2009 presented the mildest clinical symptoms. We have also discovered that disease severity (by clinical illness and histopathology) correlated with influenza specific antibody response but not viral replication in the upper respiratory tract. H1N1pdm induced the highest and most rapid antibody response followed by seasonal A/H3N2, seasonal A/H1N1 and seasonal influenza B (with B/Hubei-Wujiagang/158/2009 inducing the weakest response). Our study is the first to compare the clinical features of multiple circulating influenza strains in ferrets. These findings will help to characterize the clinical pictures of specific influenza strains as well as give insights into the development and administration of appropriate influenza therapeutics.

## Introduction

Influenza is a RNA virus of the orthomyxoviridae family and causes respiratory infections among birds and mammals [1], [2]. Since the first documented influenza pandemic in 1918–20, there have been six influenza pandemics that have resulted in millions of deaths worldwide, where the fatality rate can reach up to 2.0% as in the case of pandemic 1918–20 [2]–[5]. In addition to pandemic influenza outbreaks, seasonal influenza epidemics occur in regions of the globe annually and result in approximately three to five million cases of severe illness, and 250,000 to 500,000 deaths each year [6]. Influenza infections are caused by influenza A, B or C species where most cases are due to A or B viruses [2], [4], [7]. Specifically, influenza A can be classified into several subtypes according to the specific combination of its two surface molecules, haemagglutinin (HA) and neuraminidase (NA). There are 16 HA isotypes (H1–H16) and 9 NA isotypes (N1–N9) [2], [8]. Influenza B has only one subtype [9].

The high diversity of influenza A is attributed to genetic mutation and gene reassortment which occurs by genome segment swapping among various subtypes while infecting the same host at the same time [3], [5], [10]. Throughout history, several influenza pandemics including pandemic 2009 H1N1 (H1N1pdm) resulted from gene reassortment among several influenza subtype lineages while seasonal epidemics occurred due to rapid gene mutation [2], [3], [5], [10], [11]. Influenza A/H3N2 and A/H1N1 are the current circulating seasonal subtypes [7], [12]. Interestingly, since early 2009 to 2010, the A/H1N1 subtype was responsible for both seasonal influenza infections and a pandemic influenza outbreak but the actual causative strains for each respective A/H1N1 infection were genetically unrelated [13]. Strain variation derived from either of the two mechanisms can cause significant differences in infectivity despite the strains being classified in the same subtype. In contrast, influenza B has a slower mutation rate [14], [15], and due to its limited host tropism, gene reassortment is rare [16], [17]. Consequently, there are much fewer genetic (and antigenic) influenza B variants. To date, all influenza B infection outbreaks are only found as regional epidemics [4], [7]. Various influenza subtypes as well as genetic alterations can cause different epidemics and trigger immune responses upon infection. However, the association between human clinical outcomes with the influenza genetic background is not clear because the infections are often complicated by secondary factors such as age differences and underlying medical conditions [18], [19].

The most common clinical responses caused by influenza include a sudden onset of fever, sneezing, dry cough, rhinorrhea, myalgia and lethargy [2]–[4]. These symptoms result from the body's inflammatory and defence mechanisms during influenza infection and are often used as indices to compare disease severity in humans between influenza subtypes [3], [5], [7], [18]–[21]. After exposure to the virus, the body generates antibodies against the specific influenza strain it has encountered. Anti-HA being the most important humoral immune response creates immunity to the same or homologous influenza strains [2]. Some antibodies have neutralizing ability to interfere with viral entry to the host cell. Haemagglutination inhibition (HI) and microneutralization (MN) are the two standard assays to measure anti-HA and influenza neutralizing antibody levels respectively [22].

Since 2009, the predominant influenza strain shifted from H1N1pdm to the new A/H3N2 and Influenza B strains in several regions of the globe [23]. To date, little is known about the new influenza viruses and their clinical and immunopathology correlation. In this study, we investigated and compared clinical characteristics caused by the most current influenza strains including A/H1N1 (H1N1pdm and former seasonal A/H1N1), seasonal A/H3N2 and seasonal Influenza B in ferrets during a 14-day time course to determine the clinical picture of each influenza strain infection. Ferrets are a suitable animal model for influenza infection because they display similar flu-like symptoms and immune responses to humans [24]–[31]. Specifically, daily measurements in body temperature, body weight, nasal discharge, sneezing, and animal inactivity score upon infection were analysed. We have also evaluated influenza specific antibody responses, histopathology and viral replication for various influenza strains infections. Our findings will help clarify the influenza clinical pictures of the current circulating strains and the parallel immunopathology association. The work here will facilitate the development of future influenza diagnostics and anti-viral treatments.

## Results

### Clinical comparison of seasonal A/H1N1 influenza strains showed A/Solomon Islands/03/2006 to have severe clinical disease

Before the emergence of H1N1pdm, seasonal A/H1N1 was a major concern circulating in many regions of the world [7], [12], [13]. Although, the emergence of H1N1pdm virus seemed to preside after 2009 [30], the clinical examination of seasonal A/H1N1 infection is important not only to determine the clinical trends of typical seasonal A/H1N1 infections but also to differentiate the immunopathological responses between the pandemic and seasonal A/H1N1 infections.

**Figure 1:**
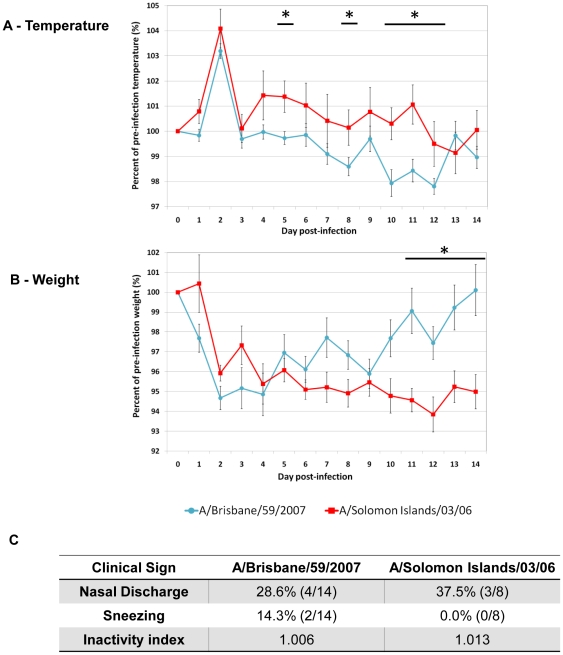
A/Solomon Islands/03/2006 seasonal A/H1N1 subtype showed more severe clinical disease compared to A/Brisbane/59/2007. Clinical signs of A/Brisbane/59/2007 (N = 14, 10^6^ EID_50_) or A/Solomon Islands/03/2006 (N = 8, 10^6^ EID_50_) infected ferrets were measured daily over 14 days. Temperature A) and weight B) were recorded daily until end day and are expressed as percentage relative to the pre-infection level at Day 0. Percent nasal discharge, percent sneezing and inactivity index C) were observed daily and the highest percentages and fractions of infected ferrets displaying symptoms are shown. Physical inactivity index measures the degree to which ferrets respond to environmental stimuli with a basal level of 1.000. Error bars represent standard error of the mean. *p<0.05 from Student's t-test.

We investigated two seasonal A/H1N1 influenza viruses, A/Solomon Islands/03/2006 (Sol/03) and A/Brisbane/59/2007 (Bris/59). Both seasonal A/H1N1 viruses were recommended for the A/H1N1 component of seasonal influenza vaccines in 2008 and 2009 [32]. Ferrets were infected with either seasonal A/H1N1 virus at 10^6^ 50% egg infectious dosage (EID_50_) and body temperature, weight, inactivity level, sneezing and nasal discharge from each group were observed daily until Day 14 post-infection (pI). We have also examined uninfected ferrets for 14 days as additional healthy control. Uninfected ferrets did not display fever (temperature never being 1% above the baseline) and weight loss. Furthermore, no sign of nasal discharge, sneezing, and inactivity was observed (Figure S1). In contrast, both seasonal A/H1N1 influenza viruses gave an immediate spike in body temperature at Day 2 pI with Sol/03 infection displaying a more rapid and higher response (Figure 1A). In addition, Sol/03 had a smaller second rise in temperature from Day 3 to 8 pI (Day 5 and 8 pI with *p_Anova_*<0.05). Bris/59 recovered to the pre-infection level after Day 3 although it gave a temperature decline from Day 9 to 13. In contrast to body temperature, Bris/59 animals lost weight more rapidly than Sol/03 animals (although not significant). However, Sol/03 animals did not show weight recovery during the observation period where Bris/59 animals recovered (Figure 1B, Day 11 to 14 pI, *p_Anova_*<0.05). More Sol/03 animals exhibited nasal discharge and appeared lethargic compared to Bris/59 animals, although no sneezing was observed from Sol/03 animals (Panel 1C). In summary, Sol/03 infected animals experienced prolonged weight loss and fever, higher incidence of nasal discharge and were more lethargic in comparison to the Bris/59 infected animals. Sol/03 infection had a more severe effect on ferrets.

### A/California/07/2009 infection caused a more severe clinical disease compared to other H1N1pdm strains

H1N1pdm influenza virus emerged in early 2009 spreading quickly around the globe where several strains were isolated from various locations [13]. A/California/07/2009 (Cal/07) and A/Mexico/4108/2009 (Mex/4108) are two of the earliest isolates identified and have been shown to be serologically and genetically related. A/Utah/20/2009 (Utah/20) was grown in tissue culture and is serologically related to Cal/07 (data not shown). A/South Carolina/2/2010 (SC/2) was collected in 2010 and it belongs to H1N1pdm lineage which is also serologically related to Cal/07 (data not shown). Here we sought to compare the physiological impact of Cal/07, Mex/4108, Utah/20, and SC/2 infections in ferrets by observing the clinical features following infection.

**Figure 2:**
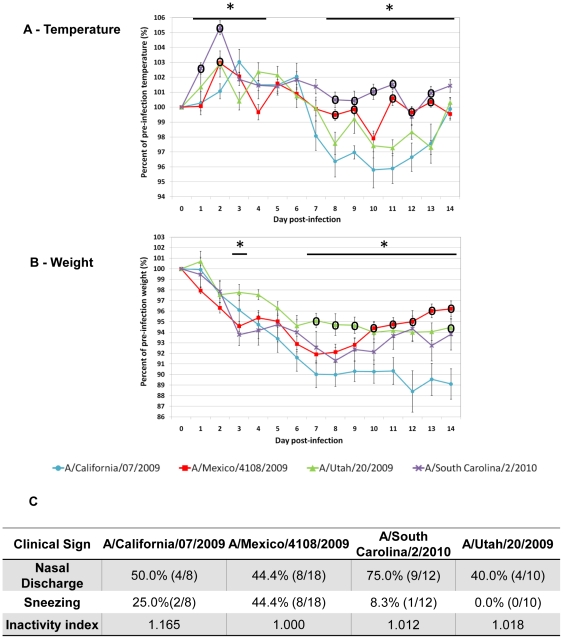
A/California/07/2009 infection gave the most severe clinical symptoms of H1N1pdm virus strains. Clinical signs of ferrets infected with A/Mexico/4108/2009 (N = 18, 10^5^ EID_50_), A/California/07/2009 (N = 8, 10^4^ EID_50_), A/South Carolina/2/2010 (N = 12, 10^5^ EID_50_), or A/Utah/20/2009 (N = 10, 10^4.3^ TCID_50_) were measured over a 14-day time course. Body temperature A) and weight B) were recorded daily until Day 14 pI. Both measurements are expressed as percentage relative to the pre-infection level at Day 0. C) summarises percent nasal discharge, percent sneezing and inactivity index. These signs were observed daily and the highest percentages and fractions of infected ferrets displaying symptoms are shown. Physical inactivity index measures the degree to which ferrets respond to environmental stimuli with a basal level of 1.000. Error bars represent standard error of the mean. *p_Anova_<0.05 and °significant difference from A/California/07/2009 by Bonferroni-holm test (post-hoc analysis).

Ferrets were infected as above except with Mex/4108 or SC/2 at 10^5^ EID_50_, Cal/07 at 10^4^ EID_50_ or Utah/20 at 10^4.3^ 50% tissue culture infectious dosage (TCID_50_). Furthermore, to show infection at the same dose, we included a clinical data comparison between H1N1pdm infections (Figure S2) at 10^6^ EID_50_. Clinical signs were observed daily for 14 days pI. Among the H1N1pdm strains from 2009, although ferrets infected by Cal/07 showed one-day-delayed temperature rise, Cal/07 induced the longest duration of fever while Mex/4018 and Utah/20 induced a biphasic pattern of fever (Figure 2A). Cal/07 infected animals also experienced the most severe weight loss among all four strains. SC/2 infection induced a substantial level of fever in the animals at Day 2 and the temperature gradually reached near the basal level at Day 8 pI. Ferrets infected with Mex/4108 recovered the earliest where their body temperatures returned to the pre-infection level and body weights started to recover after Day 7 pI (Figure 2A and 2B). Conversely, the weight of Cal/07 and Utah/20 ferrets did not recover during the observation period and there was a profound body temperature decline after Day 7 pI. The average weights from the SC/2 infected animals showed recovery but did not reach the pre-infection level at Day 14 pI. SC/2 infected animals had the highest incidence of nasal discharge followed by Cal/07 infected cohorts which appeared the most lethargic (highest inactivity index) (Panel 2C). Mex/4108 infected animals had the highest observed incidence of sneezing followed by Cal/07 infected animals. No sneezing was observed from Utah/20 infected ferrets. Taken together these results showed Cal/07 had the most severe physiological impact on ferrets, despite the genetic and serological homology among the four pandemic strains.

### A/Perth/16/2009 infection gave more severe clinical symptoms compared to other A/H3N2 seasonal influenza strains

A/H3N2 virus is also a common seasonal influenza A subtype in current circulation [32]. Four strains of A/H3N2 were examined in this study: A/Brisbane/10/2007 (Bris/10), A/Wisconsin/15/2009 (Wis/15), A/Perth/16/2009 (Perth/16), and A/Victoria/210/2009 (Vic/210). These strains have been recommended for previous and current year influenza vaccine preparations demonstrating that they are serologically important and have geographical prevalence [32].

**Figure 3:**
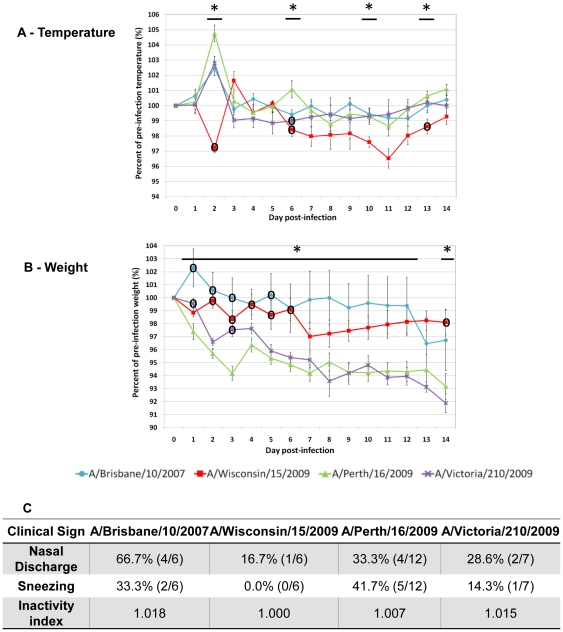
A/Perth/16/2009 infection was more severe than other A/H3N2 seasonal influenza strains. Ferrets were infected with A/Brisbane/10/2007 (N = 6), A/Wisconsin/15/2009 (N = 6), A/Perth/16/2009 (N = 12), or A/Victoria/210/2009 (N = 10 and N = 7 after Day 8 pI) and clinical signs were measured daily over a 14-day time course. The same dosage (10^6^ EID_50_) was used for all three infections. Body temperature A) and body weight B) were recorded daily to Day 14 pI. Both measurements are expressed as percentage relative to the pre-infection level at Day 0. Panel C summarises percent nasal discharge, percent sneezing and inactivity index. Percent nasal discharge and percent sneezing were observed daily and the highest percentages and fractions of infected ferrets displaying symptoms are shown. Physical inactivity index measures the degree to which ferrets respond to environmental stimuli with a basal level of 1.000. Error bars represent standard error of the mean. *p_Anova_<0.05 and °significant difference from A/Perth/16/2009 by Bonferroni-holm test (post-hoc analysis).

Ferrets were infected as above except with A/H3N2 influenza strains at 10^6^ EID_50_. Daily clinical data were collected until Day 14 pI. Perth/16, Vic/210, and Bris/10 infection resulted in an immediate and transient fever at Day 2 pI where the intensity of Perth/16 fever was similar to the seasonal A/H1N1 fevers (Figure 3A and 1A). Conversely, the temperature elevation induced by Wis/15 was delayed one day following a decline on Day 2 pI. Although Wis/15 fever (Day 3 pI) was the mildest among the four infections, the increase in body temperature was similar to Perth/16 infection (Figure 3A). For all the A/H3N2 infections, animal body temperature recovered to the pre-infection level one day following the initial fever, except for Wis/15 infected animals which experienced a long duration of hypothermia from Day 5 to Day 14 pI. Perth/16 and Vic/210 infections led to substantial weight loss where animal weights did not recover throughout the observed time course (Figure 3B). Wis/15 infected animals lost weight gradually until Day 7 and then slowly recovered. In contrast to the other A/H3N2 viruses, Bris/10 infection began with a weight-gaining spike at Day 2 pI and weight loss did not occur until Day 13. This response was not common among all the influenza viruses investigated. In regard to body temperature and weight fluctuation, Bris/10 infection was the mildest among the four A/H3N2 strains, although it rendered the highest incidence of nasal discharge. Furthermore, Bris/10 animals appeared to be the most lethargic followed by the Vic/210, Perth/16 and Wis/15 infected animals. Perth/16 infection caused the most incidence of sneezing which was not observed in Wis/15 infection (Panel 3C). Taken together the results showed that Bris/10 and Wis/15 infection were milder but instigated different physiological conditions. Moreover, Perth/16 infection was the most severe infection based on our studied clinical traits.

### Clinical characteristics of B/Florida/04/2006 and B/Hubei-Wujiagang/158/2009 are similar but distinct from B/Brisbane/60/2008 infection

Influenza B viruses belong to a species of influenza that is antigenically and genetically distant from influenza A viruses. B infections are not as common as influenza A virus infections and the host range is limited, with humans being the primary hosts [9]. Ferrets infected by influenza B also displayed similar flu-like symptoms and immune responses to humans, therefore they are a suitable model to study influenza B immunopathology [26]. We examined clinical characteristics of three strains of B viruses: B/Brisbane/60/2008 (B/Bris), B/Florida/04/2006 (B/Fla), and B/Hubei-Wujiagang/158/2009 (B/Hubei) which have been recommended for previous and/or current influenza vaccine inclusion [32].

**Figure 4:**
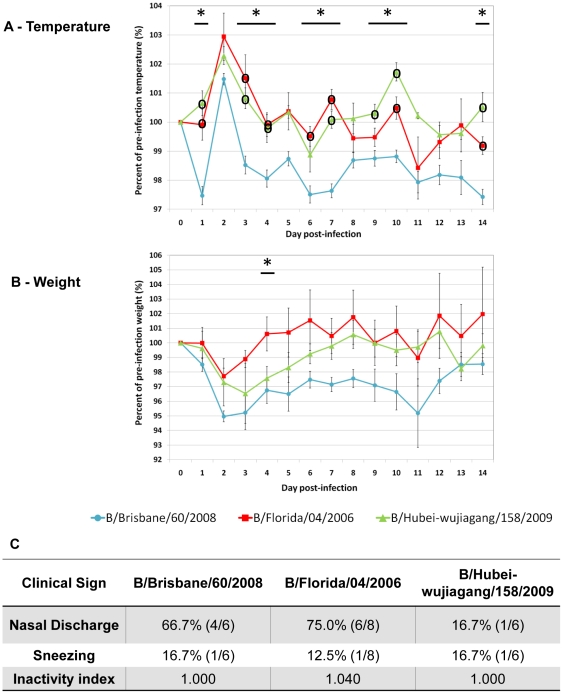
Comparison of clinical features among influenza B subtypes showed B/Brisbane/60/2008 infection to give divergent illness. Clinical signs of ferrets infected with B/Brisbane/60/2008 (N = 6), B/Florida/04/2006 (N = 8), or B/Hubei-Wujiagang/158/2009 (N = 6) were measured daily in a 14-day time course. The same dosage (10^6^ EID_50_) was used for all three infections. Body temperature A) and body weight B) were recorded daily for 14 days. Both measurements are expressed as percentage relative to the pre-infection level at Day 0. C) summarises qualitative clinical signs: percent nasal discharge and percent sneezing were observed daily and the highest percentages and fractions of infected ferrets displaying symptoms are shown. Physical inactivity index measures the degree to which ferrets respond to environmental stimuli with a basal level of 1.000. Error bars represent standard error of the mean. *p_Anova_<0.05 and °significant difference from B/Brisbane/60/2008 by Bonferroni-holm test (post-hoc analysis).

Ferrets were infected similarly as above except with influenza B strains at 10^6^ EID_50_ and daily clinical data was observed until Day 14 pI. All three infections induced fever on Day 2 pI (Figure 4A). On Day 1, B/Bris initiated a temperature decline before a rise. In opposition, B/Fla and B/Hubei gradually elevated body temperature until Day 2. B/Bris fever was the mildest and most transient although it gave the highest temperature increase from Day 1 to Day 2 pI. Following Day 3 pI, body temperature from B/Bris infected animals dropped below pre-infection level and did not recover (Figure 4A). Both B/Fla and B/Hubei fever ended on Day 4 pI and then oscillated above and below pre-infection level until Day 14 pI. In contrast to body temperature trends, B/Bris animals lost their weights rapidly starting from Day 1 pI. Conversely, B/Fla and B/Hubei animals did not experience weight loss until Day 2 pI (Figure 4B). B/Bris induced the most weight loss; furthermore there was a second weight drop from Day 8 to Day 11 pI following the first recovery and weights did not recover to the basal level on Day 14 pI. Weight loss in B/Fla and B/Hubei animals was less severe. B/Fla animals reached peak weight loss Day 2 pI and recovered to the pre-infection levels after Day 4. B/Hubei animals had highest weight loss at Day 3 and gradually recovered after Day 7 (Figure 4B). For both B/Fla and B/Hubei, body weight fluctuated around pre-infection level after weight recovery. Among three influenza B viruses, only B/Fla affected ferret activity response (Panel 4C). B/Fla induced the highest and B/Hubei induced the lowest nasal discharge incidence. However, the lowest incidence of sneezing was observed in B/Fla infection although the difference was not significant. In summary, although the three influenza B strains provoked different illness conditions, we found the clinical pattern of B/Bris infection (more weight loss, slower weight recovery and lower fever) to be different than that of B/Fla and B/Hubei. B/Fla was similar to B/Hubei infection where B/Bris seemed to evoke a divergent illness.

### Clinical characteristics of various influenza subtype infections

After the clinical results were compared among each strain of the same influenza subtype, we grouped the data accordingly: H1N1pdm, seasonal A/H1N1, seasonal A/H3N2, and seasonal Influenza B viruses. The grouped data was then compared against the other subtypes to identify the trends among the subtypes.

**Figure 5:**
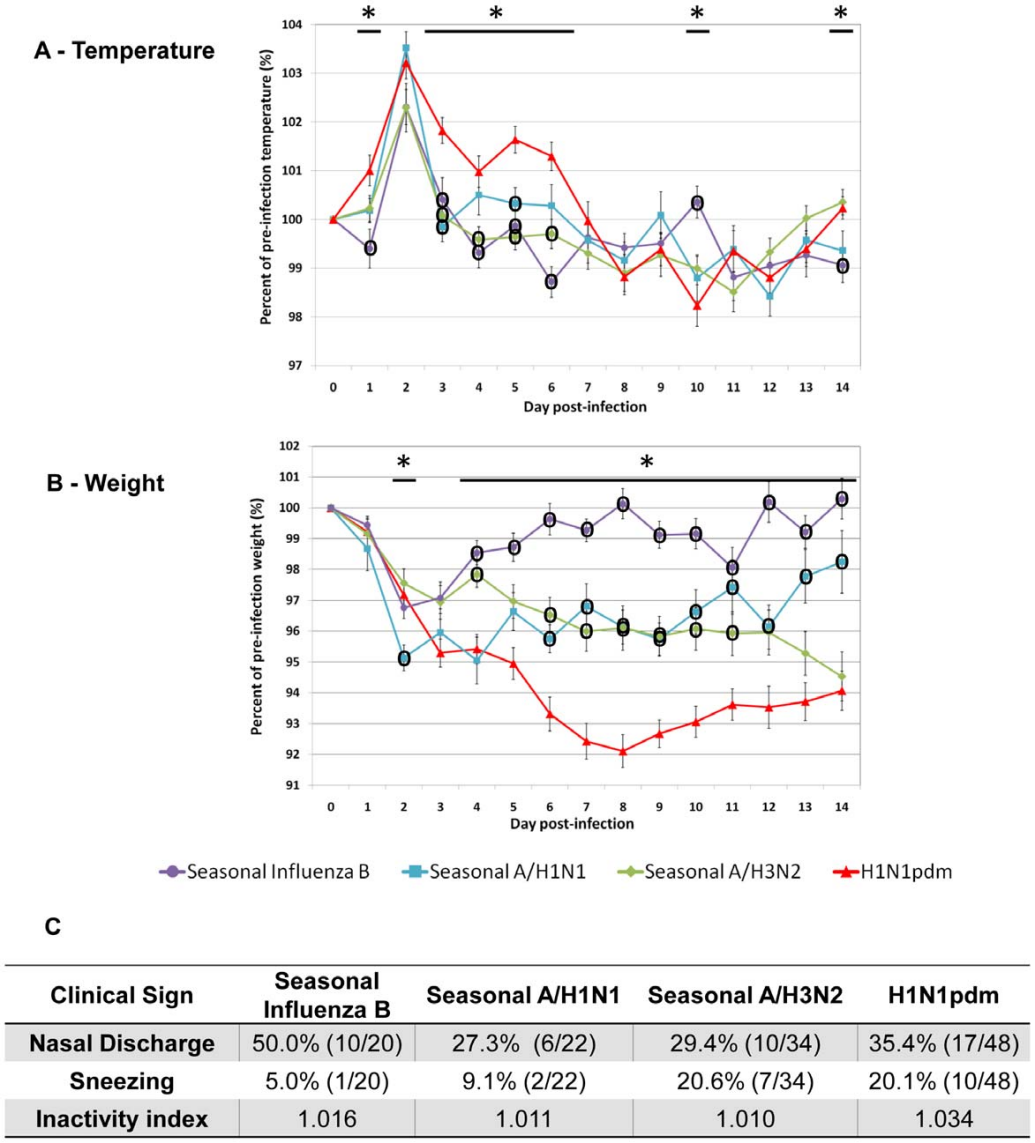
Influenza A/H1N1, A/H3N2, and B infections in ferrets gives distinct clinical trends. Ferret clinical data from H1N1pdm (N = 48), seasonal A/H1N1 (N = 22), seasonal A/H3N2 (N = 34 and N = 31 after Day 8 pI), and seasonal influenza B (N = 20) infections were combined respectively for inter-group comparison. The combined body temperature A) and body weight B) are expressed as percentage relative to the pre-infection level at Day 0. Percent nasal discharge, percent sneezing, and physical inactivity index are recalculated for each group and summarized in C). Amount of animals displaying nasal discharge and sneezing from each group were combined and calculated as percentage for each day. Only the highest percentages and fractions from each group are shown. Physical inactivity index is the pooled measure of which infected ferrets by each group respond to environmental stimuli with a basal level of 1.000. Error bars stand for standard error of the mean. *p_Anova_<0.05 and °significant difference from H1N1pdm by Bonferroni-holm test (post-hoc analysis).

In general, influenza induced fever in ferrets at Day 2 pI (Figure 5A). Seasonal A/H1N1 provoked the highest fever spike (although not significant) where H1N1pdm induced the longest fever duration (Day 1 to Day 6 pI with most days *p_Anova_*<0.05). The fever pattern of seasonal A/H3N2 and B infections were similar; both were mild and recovered after Day 3. Fever in all season infections was transient and body temperature fluctuated above and below pre-infection levels following the initial recovery. Conversely, H1N1pdm animals experienced hypothermia from Day 7 to Day 10 and gradually recovered by Day 14 (Figure 5A).

The majority of infected animals experienced weight loss Day 1 pI (Figure 5B). Weight loss in influenza B infection was the modest and peaked on Day 2 then slowly recovered. Seasonal A/H3N2 infected animals gradually lost weight and did not display weight recovery over the observation period. Seasonal A/H1N1 animals suffered an early and substantial weight loss that continued from Day 1 to Day 4. Although their body weights did not reach the pre-infection level by Day 14 pI, they gradually recuperated throughout the period. H1N1pdm animals underwent an extensive weight loss during the entire period. Their weights decreased consistently until Day 8 pI (Figure 5B) and the weight recovery was the lowest among all the studied influenza species (*p_Anova_*<0.05).

H1N1pdm animals appeared the most lethargic while the inactivity levels between the three seasonal influenza infected animals were similar (Panel 5C). Among the seasonal infections, A/H3N2 viruses induced the highest incidence of sneezing. Even though nasal discharge was observed in all influenza infections, influenza B infection gave the highest percentage of animals showing nasal discharge. Taking into account all the investigated clinical parameters, H1N1pdm had the most profound physiological impact on ferrets due to the longest weight loss period, minimal weight recovery, longest duration of fever, the highest lethargy and prominent sneezing and nasal discharge. Seasonal viruses induced differential degrees of clinical features while influenza B viruses caused the mildest illness. In addition, further analysis was performed between subtype infections (Figure S3) all at 10^6^EID_50_.

### Viral replication of representative influenza strains in the upper respiratory tract of infected ferrets

**Figure 6:**
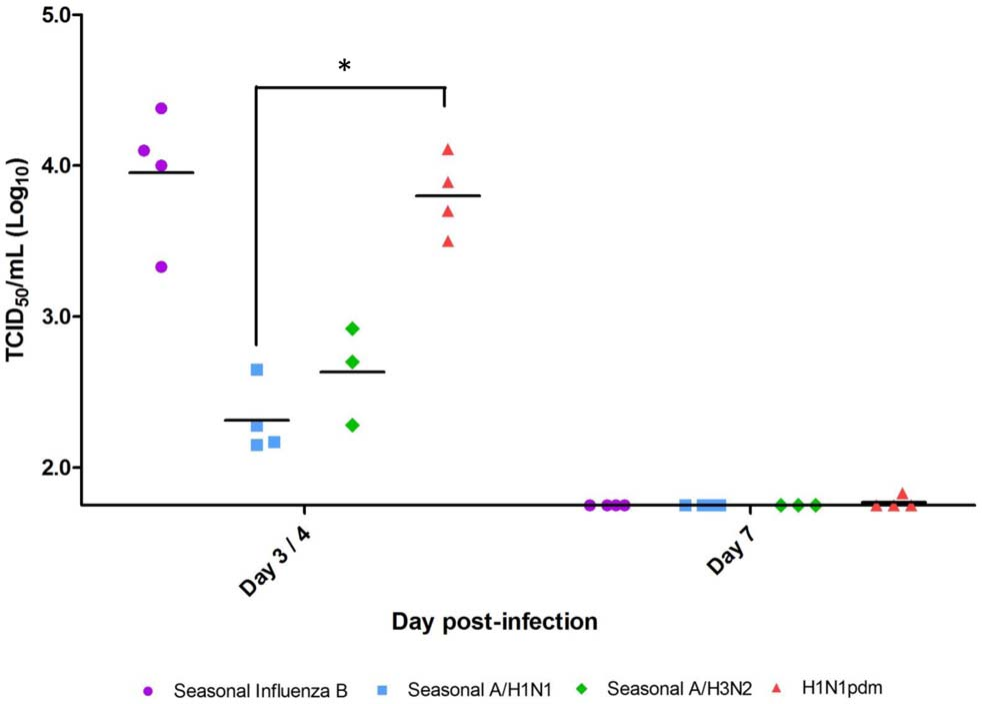
Influenza viral replication in the upper respiratory tract of ferrets following intranasal infection. One most recent circulating representative strain for each subtype was chosen as follows: Mex/4108 for H1N1pdm, Bris/59 for seasonal A/H1N1, Vic/210 for seasonal A/H3N2, and B/Hubei for seasonal influenza B. All nasal wash samples from infected ferrets were collected on Day 3 and Day 7 pI except for Vic/210 infection (Day 4 and Day 7 pI) and titrated on MDCK cells (TCID_50_/mL). Statistical analysis was only conducted on samples collected on the same day pI. *p_Anova_<0.005 and significant difference was found between H1N1pdm with seasonal H1N1 by Bonferroni-holm test (post-hoc analysis).

We next sought to determine if disease severity was associated with viral replication, specifically the viral titer in the upper respiratory tract of infected ferrets. One strain was chosen as the representative virus for each subtype. Nasal washes were collected on Day 3 or 4 and Day 7 pI and titrated on Madin-Darby Canine Kidney (MDCK) cells to measure viral titer. All the representative strains were detected in either Day 3 or Day 4 but were cleared by Day 7 pI (Figure 6). In addition, Influenza B virus titer was found at the similar level to H1N1pdm at Day 3 pI and significantly higher (*p_Anova_*<0.005) than seasonal A/H1N1.

### Differential antibody responses induced by various influenza strains

**Figure 7:**
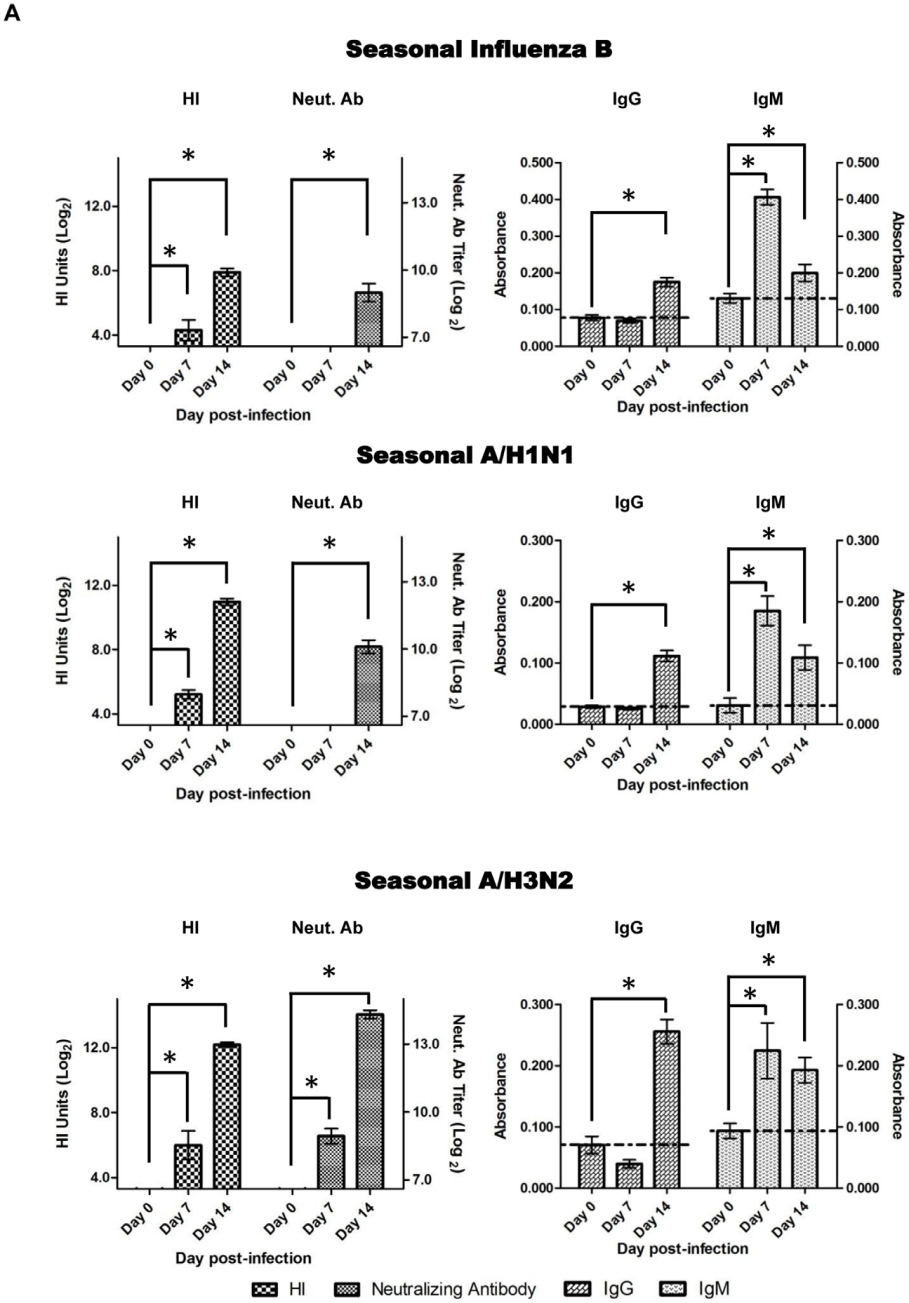
Influenza induced antibody responses mirror specific influenza strain illness. A) Time course of influenza induced antibody responses for various subtypes. Ferret anti-sera taken at the indicated days pI were used to measure HI, neutralizing antibodies and relative IgG or IgM levels. Student's t-test was conducted to compare with results on Day 0, *p<0.05. B) Day 14 pI HI titers from individual animals infected by corresponding influenza strains. HIs of A/Mexico/4108/2009 infected animal sera was used to compare with HIs of other strains using Student's t-test. *p<0.005, **p<0.000005.

The correlation between the antibody responses with intranasal viral titre and infection severity is unclear. We found that Day 7 pI, HI titers against corresponding H1N1pdm was significantly higher (*p*<0.05) than HI titers against the other corresponding seasonal influenza subtypes (Figure 7A and Table 1). The titer level from pandemic infection was elevated more rapidly than the seasonal subtypes. The similar trend was also found in MN assays where high level of neutralizing antibody titers stimulated by H1N1pdm infection was the most rapidly raised on Day 7 pI (Figure 7A and Table 1). We could not detect neutralizing antibodies for seasonal A/H1N1 and influenza B on Day 7. Antibody response induced by B/Hubei infection was significantly lower than those of other infections (Figure 7A & 7B). We found that the weak response from B/Hubei was not accompanied by an impaired antibody isotype class switching event (Figure 7A). There was no significant difference in the class switching process induced by various influenza subtypes. Anti-influenza ferret IgM was the primary antibody species on Day 7 and gradually replaced by anti-influenza IgG on Day 14 pI (Figure 7A). However, IgM species induced by influenza A infection had a longer half life in the blood stream than influenza B induced IgM. We also found HI levels were equivalent among each subtype of influenza A species but HI levels were significantly different between strains of influenza B species (Figure 7B). In summary, influenza specific antibody response induced by H1N1pdm infection has the highest magnitude and was the most rapid, followed by seasonal A/H3N2, seasonal A/H1N1, and seasonal influenza B (Figure 7A and 7B).

**Table 1:** HI and neutralizing antibody titer kinetics post-influenza infections.

	Average HI Titer‡			Average Neut. Ab Titer‡		
Day pI	Day 0	Day 7	Day 14	Day 0	Day 7	Day 14
**H1N1pdm**	<3.3	11.1	13.1	<6.6	12.9	13.4
**Seasonal H1N1**	<3.3	5.2*	11*	<6.6	<6.6*	10.1*
**Seasonal H3N2**	<3.3	6*	12.2*	<6.6	8.9*	14.3
**Influenza B**	<3.3	4.3*	7.9*	<6.6	<6.6*	9*

‡ Titer unit in Log_2_.

*Comparison of HI and neutralizing antibody titers of seasonal viruses to H1N1pdm on the indicated days pI using Student's t-test, *p*<0.05.

### Differential histopathology in the ferret infected lungs

**Figure 8:**
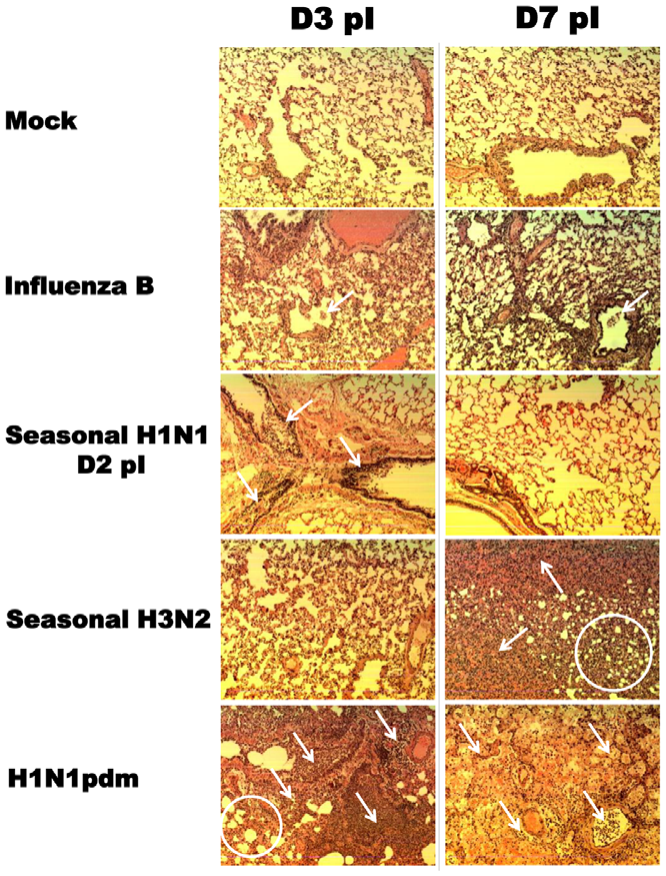
Histopathology of influenza subtype infected lung tissues by H&E stain. The representative strain for each subtype was selected as follows: Mex/4108 for H1N1pdm, Bris/59 for seasonal H1N1, Per/16 for seasonal H3N2, and B/Wis (same influenza B lineage as B/Hubei) for seasonal influenza B. All tissue samples from infected ferrets were collected on Day 3 and Day 7 pI except for Bris/59 infection (Day 2 and Day 7 pI due to facility scheduling). Representative slides are shown with 10× magnification.

In order to strengthen the clinical observations, we preformed a time course lung histopathology study during influenza infection. Ferrets were infected with representative strains from each influenza subtype or mock infected as negative control. In the mock control group, both bronchiolar and alveolar spaces were clear and no lesions were observed in the lungs on either Day 3 or 7 pI. However, leukocyte plugs were found in several bronchiolar lumens with necrotizing bronchiolar walls and sloughing of epithelial cells in the seasonal H1N1 (Day 2 pI) and influenza B (Day 3 pI) infected lungs (occasionally in the alveolar space), indicated by the white arrows (Figure 8). The majority of leukocyte plugs were cleared from the lumens with minimal epithelial shedding on Day 7 pI for both infections. In H3N2 infected lungs, no leukocyte plugs or lesions were found in the air space on Day 3 pI, similar to the mock control. However, alveolar consolidation and pulmonary haemorrhages progressively appeared in a number of areas in the H3N2 infected lobes on Day 7 pI evidenced by presences of massive number of erythrocytes (with occasional neutrophils) and interstitial edema in the bronchiolar and alveolar spaces, indicated by the white arrows. Hyperplasia of the alveolar epithelium was also observed (the enclosed area of the white circle) on Day 7 pI. H1N1pdm Infected lungs showed acute and severe inflammation on Day 3 pI evidenced by necrotizing bronchiolitis (sloughing of epithelial lining and leukocyte plugs) and alveolar consolidations and haemorrhage (massive number of leukocytes and edema fluid in the alveolar space), indicated by the white arrows. Hyperplasia of alveolar type II pneumocytes was observed (the enclosed area of the white circle) on Day 3 pI. The pathology minimized by Day 7 pI where more leukocyte plugs and haemorrhages were present in the lungs. Moreover, profound desquamation of bronchiolar and alveolar epithelial cells caused partial or almost complete disappearance of air space wall lining.

## Discussion

This study demonstrated a broad spectrum comparison of clinical characteristics among several prevalent influenza strains that have circulated in recent years. Often the compilation of human clinical data is complicated by secondary factors such as age differences, underlying medical conditions, unknown exposing dosage and influenza strain identity in different seasons [18], [19]. Here we minimized the secondary factors by using the ferret experimental model to ensure the clinical impact of each strain was due to its own pathogenicity. From our findings, H1N1pdm infection displayed the most severe clinical outcome and required a longer recovery period. Seasonal influenza subtypes caused distinct illnesses although strain-to-strain variation existed among each subtype. Our study offers an in-depth clinical discussion of circulating influenza strains that will aid future influenza diagnostics as well as facilitate the design of potential anti-influenza treatments.

Similar to high pathogenic avian influenza H5N1, H1N1pdm can cause acute respiratory distress syndromes where severe inflammation is developed in the lung and leads to respiratory failure and death [25], [31], [33], [34]. From human clinical observations, the majority of H1N1pdm hospitalized patients had at least one previous medical condition [20], [35]. Therefore, it is unclear if the pandemic strains themselves are more virulent than the seasonal strains or the virus is synergistic with predisposed conditions. We found that H1N1pdm provoked a long duration of elevated body temperature. Interestingly, this finding was not only concurrent with a previously reported study of a different H1N1pdm strain (A/Quebec/147365/2009) [24] but was in parallel with high pathogenic avian H5N1 infection in ferrets [31]. In addition, this observation was in agreement with our previous finding where a prolonged fever was induced with Cal/07 infection [29]. The H1N1pdm infections were capable of inducing profound fever and the long duration was possibly due to sustained levels of proinflammatory cytokines that are released during infection as previously seen [25], [29]. In contrast, seasonal A/H1N1 infections induced a higher magnitude but very transient fever compared to H1N1pdm. This observation is supported by our previous study that the magnitude of Bris/59 fever was higher than Cal/07 fever at the same high dosage (10^6^ EID_50_) [29].

Weight loss was more prominent in H1N1pdm infection and animals required a longer recovery period which may have been due to tissue damage that was caused by profound cytokine production and broader tissue tropism of pandemic strains [25], [27]–[29]. These results were also supported by human clinical observations where previously healthy individuals developed severe illness by the intense inflammation in the respiratory system upon H1N1pdm infection [21], [34]. In addition, the sustained fever might have affected the animals' appetite as weight recovery did not occur until temperature returned to baseline. The difference in the clinical characteristics between seasonal and H1N1pdm infection could be explained by their distant genetic background [13]. Although, H1N1pdm caused severe illness, these strains were cleared by Day 7 pI like other seasonal viruses. B species infected animals did not show high fever and prominent weight loss, however they induced the highest incidence of nasal discharge (B/Fla and B/Bris specifically) among the 13 studied influenza strains. In addition, high viral titer found in the nasal washes of influenza B virus infected ferrets on Day 3 pI implied that influenza B replicated as efficiently as H1N1pdm in the upper respiratory tract. These observations suggested that influenza B viruses were capable of establishing an infection but failed to give ferrets severe illness. Since all strains replicated at a similar efficiency in MDCK cells (data not shown), the resulting higher titer was due to increased viral replication specifically in ferrets. The similar clinical effects of B/Fla and B/Hubei could possibly be due to close genetic background as they both belong to the B/Yamagata/16/88 lineage whereas B/Bris is closely related to B/Victoria/2/87 lineage [36]. The differential clinical outcomes from different A/H3N2 strain infections can also be related to the divergence in their genetic background [37]. These findings could add insight into the recent A/H3N2 outbreak in North America by suggesting that not only is the current seasonal A/H3N2 strains more pathogenic than the previous circulating A/H3N2 strains (e.g. Bris/10) but also previous A/H3N2 vaccination provides minimal protection against the new A/H3N2 strains. Our results support the concept that differences in genetic background among the various influenza subtypes or strains can cause distinct clinical characteristics.

Antibody response plays a central role in immunity. The specificity and quantity of antibody determine the strength of humoral defence when a secondary exposure to the same or related pathogen occurs. In this study, we found that the antibody responses correlated with the severity of influenza infections. The antibody titre from H1N1pdm infection was the highest and was induced most rapidly followed by seasonal A/H3N2, seasonal A/H1N1 and then Influenza B. This observation was concurrent with infection severity (in both clinical characteristics and histopathology). H1N1pdm induced the most severe illness (maximal weight lost, slowest weight recovery, longest fever duration and the most prominent influenza-induced pulmonary lesions and inflammation) followed by seasonal A/H3N2 (gradual weight loss throughout the time course but did not recover on Day 14 pI with deteriorating pulmonary histopathology), seasonal A/H1N1 (acute onset of weight loss with early occurrence of bronchiolitis but gradual recovery throughout the observation period) and then Influenza B infection (mild weight loss and early recovery with mild bronchiolitis). In addition, B/Hubei infection induced the least severe clinical illness and also the lowest antibody titer. Similar findings were also observed in our previous study where HI titer was higher in Cal/07 than in Bris/59 animals [29]. Similarly, it was shown that the attenuated mutants of a H1N1pdm strain, A/New York/1682/2009, induced lower neutralizing antibody levels than the wild type but induced equivalent neutralizing antibody levels when the clinical influences were similar [30]. Our results reinforced the phenomenon that illness severity is proportional to antibody response.

Since infections at varying doses are unsuitable for statistical comparison, we have included a clinical data comparison between H1N1pdm infections (Figure S2) and between subtype infections (Figure S3) all at 10^6^EID_50_. Both infections at 10^6^EID_50_ and infections at lower doses show a similar clinical trend as seen in both figures. A future study of histology should be conducted to observe the influenza replication and inflammation sites in tissues and the corresponding viral titer measurements of the upper and lower respiratory tract. These findings would demonstrate the molecular signatures of influenza infections from specific subtypes that can be paralleled with the clinical findings in this study. The influence of various risk factors such as pregnancy, obesity and age to a variety of influenza strain infections should also be assessed. In addition, this clinical data can be referred to when considering appropriate treatments and therapies for specific influenza strain infection and therefore the evaluation of anti-viral efficacy. Furthermore, when determining the influenza strain virulence and its impact on the clinical outcome, the genetic background of the host is also important. For example, Mex/4108 infection was mild in Balb/c mice but severe in C57BL/6 mice [27], [38]. Our outbred ferret model resembles the genetic diversity in humans. While the genetic background of the host differs, we were able to characterize clinical trends when the same virus was infected in multiple ferrets. Furthermore, the clinical symptoms of a certain virus were statistically significant when compared to other infections of different subtypes. Since outbred ferrets showed similar trends when infected with the same strains which significantly differed from infection with alternative strains, this clinical information can be used as an infection baseline model for human disease, as it is assumed that outbred ferrets have a similar genetic diversity as humans. This study has led the way to study influenza infection in a broad spectrum without the confounding secondary factors and the observations provide discussion for the understanding of influenza strain specific clinical features and insights for the design of future anti-influenza treatments.

## Materials and Methods

### Ethics statement

All work with animals was conducted in strict accordance with the Canadian Council of Animal Care (CCAC) guidelines. The University Health Network (UHN) has certification with the Animals for Research Act (Permit Number: #0045 and #0085 of the Ontario Ministry of Agriculture, Food and Rural Affairs) and follows NIH guidelines (OLAW #A5408-01). The animal use protocol was approved by the Animal Care Committee (ACC) of the UHN. All infections and sample collections were performed under 5% isoflurane anaesthesia and all efforts were made to minimize suffering.

### Viruses

All viruses are provided by Center for Disease Control and Prevention ([CDC], Atlanta, GA, USA) or American Type Culture Collection ([ATCC], Manassas, VA, USA); four strains of H1N1pdm (A/California/07/2009, A/Mexico/4108/2009, A/Utah/20/2009, and A/South Carolina/2/2010), two strains of seasonal A/H1N1 (A/Brisbane/59/2007 and A/Solomon Islands/03/2006), four strains of seasonal A/H3N2 (A/Brisbane/10/2007, A/Wisconsin/15/2009, A/Perth/16/2009, and A/Victoria/210/2009), and three strains of seasonal influenza B (B/Brisbane/60/2008, B/Florida/04/2006, and B/Hubei-Wujiagang/158/2009). Utah/20 was unable to propagate in embryonic chicken eggs. For the time course kinetics of viral titer in nasal wash and antibody titer determination, one representative strain from each subtype was selected: Mex/4108 for H1N1pdm, Bris/59 for seasonal A/H1N1, Vic/210 for seasonal A/H3N2, and B/Hubei for seasonal influenza B. For the histopathology time course study, the following was selected: Mex/4108 for H1N1pdm, Bris/59 for seasonal A/H1N1, Per/16 for seasonal A/H3N2, and B/Wisconsin/01/2010 (B/Wis) for seasonal influenza B. These strains are the most recent circulating strains and all infections are shown at 10^6^EID_50_. B/Wis was also provided by CDC (Atlanta, GA, USA) and belongs to the same influenza B lineage as B/Hubei. All virus work was performed in BSL-2 facility.

### Infection and ferrets monitoring

Maintenance and monitoring of ferrets upon infection as previously described [29]. Briefly, male ferrets 4–6 months old were purchased from Triple F Farms (Sayre, PA, USA) or bred in an on-site SPF ferret colony (University Health Network, Toronto, ON, Canada). Ferrets were shown to be seronegative by HI assay against circulating influenza A and B strains using the 2009–2010 and 2010–2011 WHO Influenza Reagent Kit for Identification of Influenza Isolates (WHO collaborating center for surveillance, epidemiology and control for influenza infection division) before infection. The kits contain the circulating influenza strains for the particular year, including: Cal/07 for H1N1pdm, Bris/59 for seasonal H1N1, Bris/10 for seasonal H3N2 (2009–2010), Per/16 for seasonal H3N2 (2010–2011), B/Bris for B/Victoria lineage, and B/Fla for B/Yamagata lineage. All viruses in this study were either the same strains or closely related to those in the WHO kits. ELISA was also performed on the pre-infection sera and found no reaction to the viruses in this study. Prior to infection, ferrets were randomly selected and pair-housed individually in cages contained in bioclean portable laminar-flow clean-room enclosures (Lab Products, Seaford, DE) in the BSL-2 animal holding area. Baseline body temperature and weight were measured on Day 0 for each animal. Temperatures were measured by using a subcutaneous implantable temperature transponder (BioMedic Data Systems, Inc., Seaford, DE). Upon infection, ferrets were anesthetized and infected with seasonal viruses (10^6^ EID_50_) or pandemic viruses (10^5^ EID_50_ for Mex/4108 and SC/2, 10^4^ EID_50_ for Cal/07 and 10^4.3^ TCID_50_ for Utah/20). The volume of inoculum was 1 mL for each ferret (0.5 mL in each nostril). Clinical signs (body temperature, body weight, level of activity, nasal discharge, and sneezing) were observed daily for 14 days pI. We examined animals at the same time each day for consistency. Nasal discharge includes crusty nose, mucous, and transparent exudates/fluids. The scores were calculated from the total animals displaying any nasal discharge symptom over the total number of animals. The sneezing scores were calculated from the total animals found sneezing over the total number of animals. Scores were calculated daily for 14 days and only the peak values for each infection are summarized in panel C in Figures 1,2,3,4,5. The inactivity scoring system is based on the reference Reuman *et al.*, 1989 [39] to assess the inactivity level: 0, alert and playful; 0.5, alert but playful only when stimulated; 1, alert but not playful when stimulated; 2, neither alert nor playful when stimulated. A relative inactivity index was calculated as follows: Σ_(day 1 to day 14)_[score+1]*_n_*/Σ_(day 1 to day 14)_ *n*, where *n* equals the total number of observations. A value of 1 was added to each observation unit score so that a score of 0 could be divided by a denominator, resulting in an index value of 1.0 as the minimum value. Nasal washes from the infected ferrets were collected on Day 3 or 4 and Day 7 pI in nasal wash buffer (1%BSA and 100 U/mL penicillin, 100 µg/mL streptomycin in PBS) and were stored in −80°C. In-life bleed were collected from infected ferrets Day 0, 7, and 14 pI for the representative viruses. Penicillin and streptomycin were obtained from Invitrogen Canada (Burlington, ON, Canada) and BSA was from Wisent Inc. (Saint-Bruno, QC, Canada).

### Determination of viral load

Viral replication in the upper respiratory tract was assessed by endpoint titration of nasal washes from the infected ferrets in MDCK cells (TCID_50_) using haemagglutination as the readout for positive wells as previously described [29]. Briefly, nasal washes were initially diluted 10 times in vDMEM (Dulbecco's modified Eagle's medium containing 1% BSA, 25 mM glucose, 1 mM sodium pyruvate, 4 mM glutamine, 100 U/mL penicillin, 100 µg/mL streptomycin, 50 µg/mL gentamycin and 1 µg/mL TPCK-Trypsin) followed by the half log serial dilution from 10^−1.0^ to 10^−6.5^ in quadruplicate with vDMEM on MDCKcells in 96-well flat-bottom plates (SARSTEDT, Inc., Saint-Leonard, QC, Canada). Before infection, MDCK cells were maintained in the log-phase with low-passage numbers and grew in cDMEM (DMEM containing 10% fetal bovine serum, 25 mM glucose, 1 mM sodium pyruvate, 6 mM glutamine, 1 mM non-essential amino acids, 100 U/mL penicillin, and 100 µg/mL streptomycin). The day before nasal wash incubation, 2×10^4^ MDCK cells were seeded into each well to reach 95% confluence the next day. After 2 hours incubation of nasal washes samples at 37°C, 5% CO_2_, samples were removed and replaced with fresh vDMEM and incubated for 6 days at 37°C, 5% CO_2_. On Day 6 pI, supernatants were examined for presence of virus by influenza haemagglutination of 0.5% turkey erythrocytes (Lampire® Biological Laboratories, Pipersville, PA, USA). The viral titers were determined as the reciprocal of the dilution resulting in 50% HA positivity. Viral titers are given as TCID_50_/ml for nasal washes. All cell culture reagents were obtained from Invitrogen Canada except for TPCK-Trypsin (Sigma-Aldrich Canada Ltd., Oakville, ON, Canada).

### Determination of influenza specific antibody responses

Influenza specific antibody responses from the uninfected or infected ferrets were measured by HI or MN as previously described [40]. Briefly, receptor destroying enzyme ([RDE], Accurate Chemical & Scientific Corp., Westbury, NY, USA) treated ferret anti-sera was serially diluted and HI titers were determined by the highest dilution that completely inhibited influenza haemagglutination (4HAU) of turkey erythrocytes. MN results were evaluated by enzyme-linked immunosorbent assay (ELISA). Neutralizing antibody titers were determined by the highest dilution of RDE-treated anti-sera that disrupted infection (100 TCID_50_) to MDCK cells at the reading lower than 50% signal reading measured from virus+cell and cell only controls.

### Determination of influenza specific ferret IgG/IgM isotype relative levels

The determination was based on ELISA technique as previously described [40]. Briefly, live influenza virions were coated to ELISA plates (Thermo Fisher Scientific, Rochester, NY, USA) followed by appropriate blocking and incubation of ferret anti-sera (1∶1000). After the incubation of HRP-anti-ferret IgG or IgM (1 µg/mL, Rockland Immunochemicals Inc. Gilbertsville, PA, USA), the plates were developed and read at 490 nm.

### Histopathology

Infected ferrets (10^6^ EID_50_, N = 3 per group) were sacrificed on Day 3 and 7 pI (except for Bris/59 which was sacrificed on Day 2 and 7 pI due to facility scheduling) for histopathology. PBS (diluent for all virus inocula) was used for mock infection control. Lung tissues excised from the upper and lower left lobes were formalin-fixed and paraffin embedded. Tissue slides were stained with hematoxylin/eosin for microscopic assessment.

### Statistics

One-way analysis of variance (ANOVA) was used for statistical analysis of the results in Figure 2,3,4,5,6 and Figure S3. Bonferroni-holm test was conducted for post-hoc analysis if statistically significant difference was found in comparison to the most pathogenic or distinct strain in each subtype. Student's t-test was conducted for Figure 1, 7 and Figure S2. A *p* value of ≤0.05 was considered as significant.

## Supporting Information

Figure S1 **Clinical characteristics of uninfected ferrets.** Clinical signs of uninfected ferrets (N = 6) were measured daily over 14 days in the same approach as the infected groups. Temperature A) and weight B) were recorded daily and are expressed as percentage relative to the baseline level at Day 0. Nasal discharge, sneezing and activity level C) were observed daily and the highest percentages and fractions of ferrets displaying symptoms are shown. Physical inactivity index measures the degree to which ferrets respond to environmental stimuli with a basal level of 1.000. Error bars represent standard error of the mean.
(TIF)

Figure S2 **Clinical characteristics of H1N1pdm infected ferrets at 10^6^EID_50_.** Clinical signs of ferrets infected with A/Mexico/4108/2009 (N = 8 between Day 0 to Day 6 pI, N = 5 between Day 7 to Day 14) and A/California/07/2009 (N = 8 between Day 0 to Day 6 pI, N = 6 between Day 7 to Day 9 and N = 5 between Day 10 to Day 14) were measured over a 14-day time course. Body temperature A) and weight B) were recorded daily until Day 14 pI. Both measurements are expressed as percentage relative to the pre-infection level at Day 0. C) summarises percent nasal discharge, percent sneezing and inactivity index. These signs were observed daily and the highest percentages and fractions of infected ferrets displaying symptoms are shown. Physical inactivity index measures the degree to which ferrets respond to environmental stimuli with a basal level of 1.000. Error bars represent standard error of the mean. *p<0.05 from Student's t-test.
(TIF)

Figure S3 **Clinical characteristics of influenza subtype infections in ferrets at 10^6^EID_50_.** Ferret clinical data from H1N1pdm (N = 16 between Day 0 to Day 6 pI, N = 11 between Day 7 to Day 9 and N = 10 between Day 10 to Day 14), seasonal A/H1N1 (N = 22), seasonal A/H3N2 (N = 34 and N = 31 after Day 8 pI), and seasonal influenza B (N = 20) infections were combined respectively for inter-group comparison. The combined body temperature A) and body weight B) are expressed as percentage relative to the pre-infection level at Day 0. Percent nasal discharge, percent sneezing, and physical inactivity index are recalculated for each group and summarized in C). Amount of animals displaying nasal discharge and sneezing from each group were combined and calculated as percentage for each day. Only the highest percentages and fractions from each group are shown. Physical inactivity index is the pooled measure of which infected ferrets by each group respond to environmental stimuli with a basal level of 1.000. Error bars stand for standard error of the mean. *p_Anova_<0.05 and °significant difference from H1N1pdm by Bonferroni-holm test (post-hoc analysis).
(TIF)
