# Supplementary figures and images for: Comparative Analyses of Pandemic H1N1 and Seasonal H1N1, H3N2, and Influenza B Infections Depict Distinct Clinical Pictures in Ferrets
Source: PLoS One. 2011 Nov 16;6(11):e27512. doi: 10.1371/journal.pone.0027512 (PMC3217968; doi:10.1371/journal.pone.0027512)

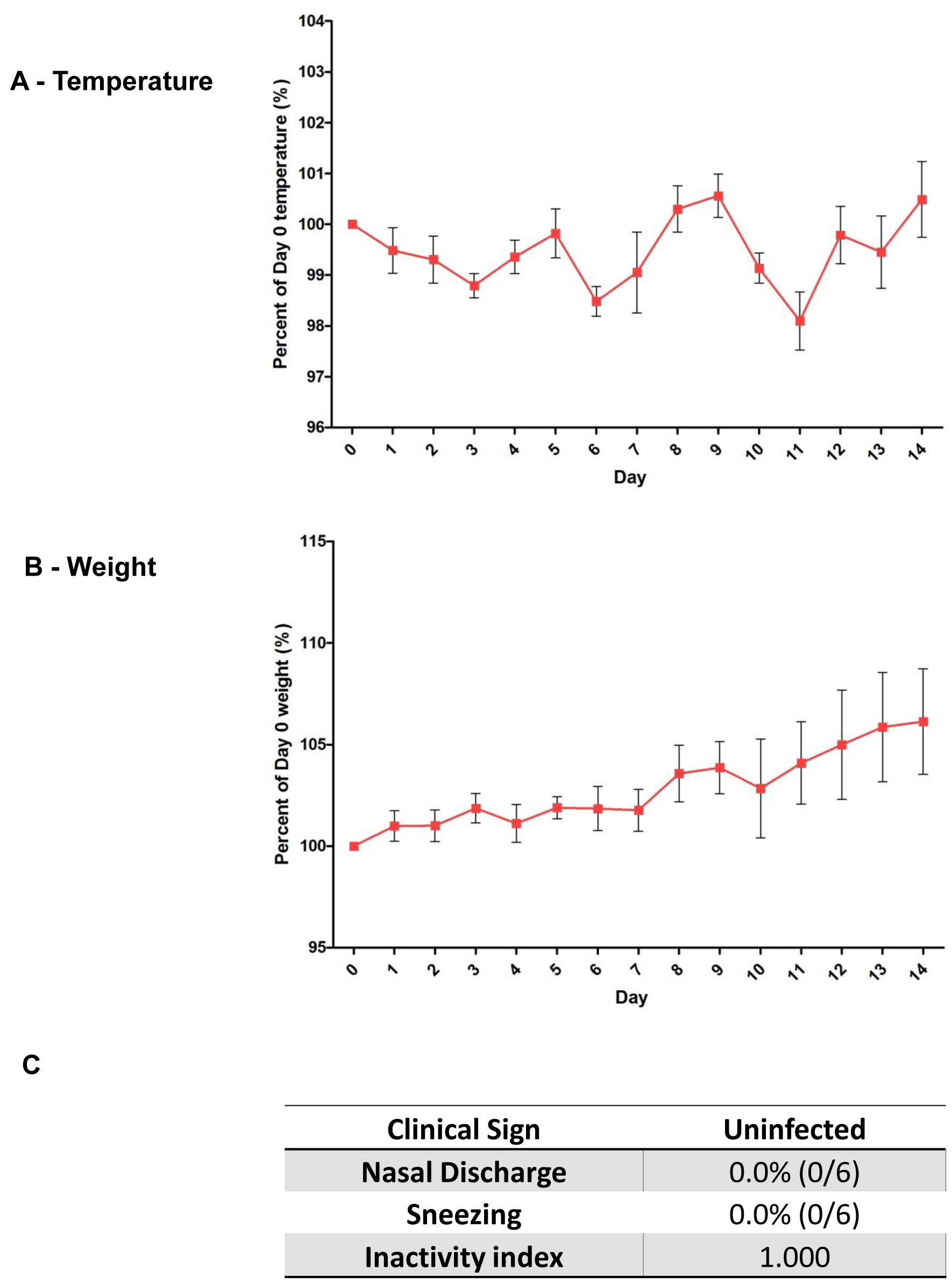

Supplement: Figure S1 — Clinical characteristics of uninfected ferrets. Clinical signs of uninfected ferrets (N = 6) were measured daily over 14 days in the same approach as the infected groups. Temperature A) and weight B) were recorded daily and are expressed as percentage relative to the baseline level at Day 0. Nasal discharge, sneezing and activity level C) were observed daily and the highest percentages and fractions of ferrets displaying symptoms are shown. Physical inactivity index measures the degree to which ferrets respond to environmental stimuli with a basal level of 1.000. Error bars represent standard error of the mean. (TIF) [file pone.0027512.s001.tif]

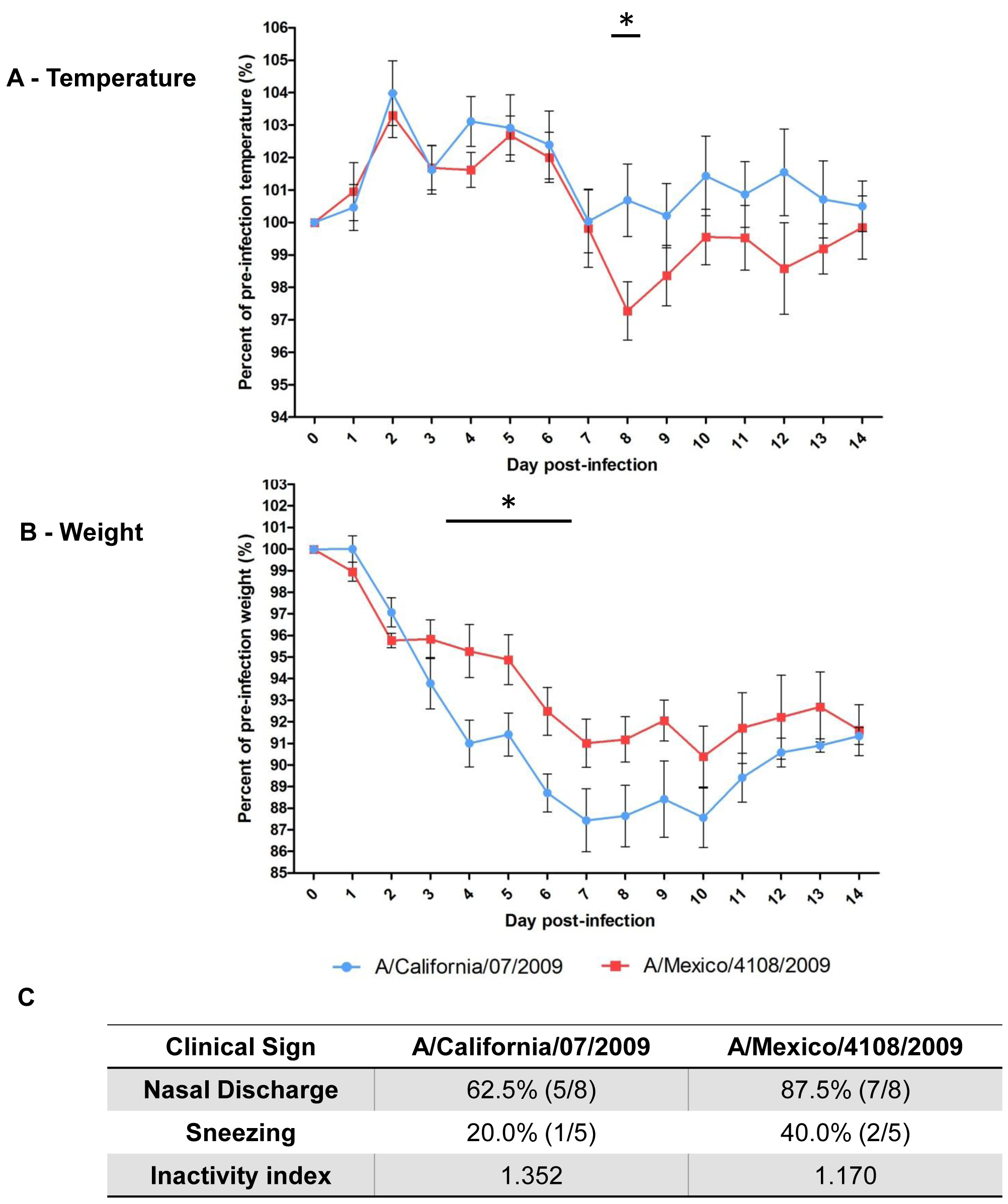

Supplement: Figure S2 — Clinical characteristics of H1N1pdm infected ferrets at 106EID50. Clinical signs of ferrets infected with A/Mexico/4108/2009 (N = 8 between Day 0 to Day 6 pI, N = 5 between Day 7 to Day 14) and A/California/07/2009 (N = 8 between Day 0 to Day 6 pI, N = 6 between Day 7 to Day 9 and N = 5 between Day 10 to Day 14) were measured over a 14-day time course. Body temperature A) and weight B) were recorded daily until Day 14 pI. Both measurements are expressed as percentage relative to the pre-infection level at Day 0. C) summarises percent nasal discharge, percent sneezing and inactivity index. These signs were observed daily and the highest percentages and fractions of infected ferrets displaying symptoms are shown. Physical inactivity index measures the degree to which ferrets respond to environmental stimuli with a basal level of 1.000. Error bars represent standard error of the mean. *p<0.05 from Student's t-test. (TIF) [file pone.0027512.s002.tif]

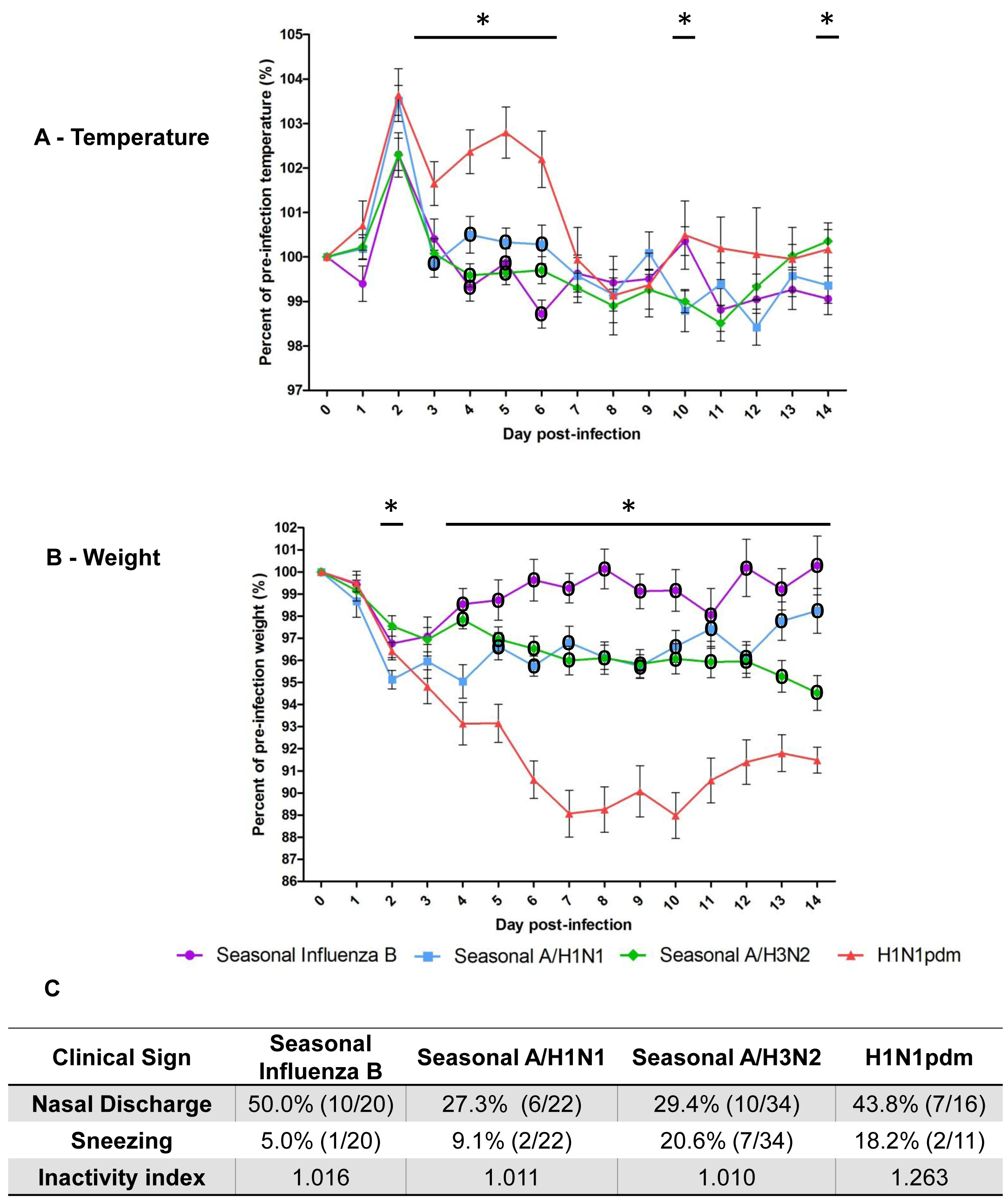

Supplement: Figure S3 — Clinical characteristics of influenza subtype infections in ferrets at 106EID50. Ferret clinical data from H1N1pdm (N = 16 between Day 0 to Day 6 pI, N = 11 between Day 7 to Day 9 and N = 10 between Day 10 to Day 14), seasonal A/H1N1 (N = 22), seasonal A/H3N2 (N = 34 and N = 31 after Day 8 pI), and seasonal influenza B (N = 20) infections were combined respectively for inter-group comparison. The combined body temperature A) and body weight B) are expressed as percentage relative to the pre-infection level at Day 0. Percent nasal discharge, percent sneezing, and physical inactivity index are recalculated for each group and summarized in C). Amount of animals displaying nasal discharge and sneezing from each group were combined and calculated as percentage for each day. Only the highest percentages and fractions from each group are shown. Physical inactivity index is the pooled measure of which infected ferrets by each group respond to environmental stimuli with a basal level of 1.000. Error bars stand for standard error of the mean. *pAnova<0.05 and °significant difference from H1N1pdm by Bonferroni-holm test (post-hoc analysis). (TIF) [file pone.0027512.s003.tif]
